# Supplementary material for: Relationships between markers of emotional and social cognition and acoustic-verbal hallucinations in children and adolescents with post-traumatic stress disorder (PTSD)
Source: PLoS One. 2025 Sep 23;20(9):e0332910. doi: 10.1371/journal.pone.0332910 (PMC12456796; doi:10.1371/journal.pone.0332910)
Supplement: Attachment — Submitted filename: Review.pdf [file pone.0332910.s002.pdf]

This study examines whether the emotional and social cognition of children and adolescents with PTSD can predict subsequent AVH, in what may be a follow-up study lasting up to seven years. This is an important issue in terms of early monitoring of the psychopathological course following trauma and may also help clarify the psychopathological underpinnings of AVH in PTSD. Suggestions and explanations are as follows.

1. **Suggested revision to phrasing of study objectives**:
   The current wording—"The primary objective of our study" and "The secondary objectives of our study"—may imply that the secondary objectives are unrelated. However, the listed secondary objectives appear to elaborate on and further specify the primary objective. Therefore, I suggest revising *"The secondary objectives of our study are"* to *"Specifically, we aim to"* or *"To this end, we further aim to"*, which would better reflect their relationship.
2. **Clarification on trauma exposure**:
   Although all participants met the DSM-5 criteria for PTSD, was there a comparison between the AVH and non-AVH groups in terms of the nature or severity of the traumatic events they experienced? Were the stressors similar across groups, or were there meaningful differences?
   Additionally, did the two groups differ in the time interval between the occurrence of the stressful event and their inclusion in the study? The current mental state of children and adolescents may also be influenced by how much time has passed since the traumatic event.
3. **Diagnostic validity and assessor qualifications**:
   Diagnostic accuracy is critical. Who administered the K-SADS-PL and the MINI-Kid (Mini International Neuropsychiatric Interview for Children and Adolescents)? Did these assessors receive formal training or hold any relevant professional licenses or certifications?
4. **WISC-IV administration**:
   Who administered the WISC-IV? Did the examiners receive standardized training or hold professional certification to ensure the reliability and validity of the assessment?
5. **Psychometric properties of measures**:
   It would strengthen the study to include more detailed information on the reliability and validity of each test used, particularly those assessing emotional and social cognition.
6. **Literature support for null findings**:
   The statement *"This study did not find significant results for markers of social cognition, contrary to data reported in the scientific literature"* implies a

contradiction with previous findings. Could the authors cite specific studies or provide references to support this comparison?

7. **IQ differences between groups**:
   The study reports no significant differences in emotional and social cognition between AVH and non-AVH groups at T0. Given that IQ was measured, were there any differences in IQ between these two groups that might account for the findings?
8. **Need for a non-PTSD comparison group**:
   The study hypothesizes that AVH emerging after PTSD may be due to impairments in emotional and social cognition, which affect individuals' interpretations of internal neural signals. However, to support this hypothesis, it may be necessary to include a matched comparison group without PTSD. If both the AVH and non-AVH groups show lower emotional and social cognition scores than the control group—despite all three groups having similar IQs—it would provide a stronger foundation for attributing AVH to post-traumatic deficits in emotional and social cognition. If such a comparison is not currently available, it is recommended to address this as a limitation of the study.
