# Supplementary material for: Relationships between markers of emotional and social cognition and acoustic-verbal hallucinations in children and adolescents with post-traumatic stress disorder (PTSD)
Source: PLoS One. 2025 Sep 23;20(9):e0332910. doi: 10.1371/journal.pone.0332910 (PMC12456796; doi:10.1371/journal.pone.0332910)
Supplement: Attachment — Submitted filename: Response to Reviewers.docx [file pone.0332910.s003.docx]

Dear Academic Editor and Reviewers,

We would like to thank you for the careful reading of our manuscript entitled *“Relationships between markers of emotional and social cognition and acoustic-verbal hallucinations in children and adolescents with post-traumatic stress disorder (PTSD)”* by Louise-Emilie Dumas, Florence Askenazy, and Arnaud Fernandez, and for the constructive comments and suggestions you have provided. We greatly appreciate the time and effort you have dedicated to helping us improve our work.

We have carefully considered each of your remarks and have revised the manuscript accordingly. In the following pages, we respond point-by-point to the comments raised by the Academic Editor and each reviewer. For clarity, each reviewer’s comment is reproduced in *italic*, followed by our detailed response and the corresponding modifications made to the manuscript. All changes in the revised version are highlighted using the “Track Changes” mode.

We believe that these revisions have strengthened the manuscript and clarified key aspects of our study, both in terms of methodology and interpretation of results.

## **Comments from the Academic Editor**

1. Style requirements

*Please ensure that your manuscript meets PLOS ONE's style requirements, including those for file naming. The PLOS ONE style templates can be found at https://journals.plos.org/plosone/s/file?id=wjVg/PLOSOne_formatting_sample_main_body.pdf and https://journals.plos.org/plosone/s/file?id=ba62/PLOSOne_formatting_sample_title_authors_affiliations.pdf*

The headings have been set in bold and size 18. Figures are presented in separate files, named as requested, with titles in bold, and are cited in the text according to the journal’s formatting guidelines. The headings have been revised to comply with the required levels. Tables have been inserted into the main text, and the data and results have been right aligned for improved readability.

2. Data availability statement

*In the online submission form, you indicated that “Summary and anonymized data would be available on the basis of a written reasonable request”. All PLOS journals now require all data underlying the findings described in their manuscript to be freely available to other researchers, either: 1. In a public repository, 2. Within the manuscript itself, or 3. Uploaded as supplementary information. This policy applies to all data except where public deposition would breach compliance with the protocol approved by your research ethics board. If your data cannot be made publicly available for ethical or legal reasons (e.g., public availability would compromise patient privacy), please explain your reasons on resubmission and your exemption request will be escalated for approval.*

In accordance with PLOS ONE’s data availability policy, we have modified the Data Availability Statement to indicate that all data underlying the findings will be provided as supplementary information files. The sentence has been modified in the manuscript to read as follows:

“All data underlying the findings described in this manuscript are provided as supplementary information files.”

3. Ethics statement location

*Your ethics statement should only appear in the Methods section of your manuscript. If your ethics statement is written in any section besides the Methods, please move it to the Methods section and delete it from any other section. Please ensure that your ethics statement is included in your manuscript, as the ethics statement entered the online submission form will not be published alongside your manuscript.*

The ethics statement has been moved to the Methods section, as requested. It now reads as follows:

“Ethical approval and consent: This study was approved by the Ethics Committee ‘Nord-Ouest III’ (France) under protocol number 17-HPNCL-03. Written informed consent was obtained from all participants and/or their legal guardians prior to inclusion in the study.”

4. Tables format

*Please include your tables as part of your main manuscript and remove the individual files. Please note that supplementary tables should remain/be uploaded as separate 'supporting information' files.*

All tables have been inserted into the main manuscript, as requested, and the individual table files have been removed. We do not have any supplementary tables to include as “supporting information” files.

5. Reviewer citation suggestions

*If the reviewer comments include a recommendation to cite specific previously published works, please review and evaluate these publications to determine whether they are relevant and should be cited. There is no requirement to cite these works unless the editor has indicated otherwise.*

Acknowledged, thank you very much.

## **Reviewer #1**

1. Inclusion of tables in the main manuscript

*To begin with, although the results are promising, I noticed that the tables were not included in the PDF file. This made it somewhat difficult to accurately interpret the findings. If possible, I would appreciate the opportunity to review the full version with the tables included.*

We thank the reviewer for pointing this out. Indeed, the tables were not included in the main manuscript but were provided in a separate file. We have now inserted all tables into the main manuscript, in accordance with the journal’s author guidelines.

2. Terminology for AVH

*I am curious as to why the term “acoustic-verbal hallucinations (AVH)” was chosen instead of directly referring to “auditory hallucinations with voice commanding.”*

We thank the reviewer for this question. We chose the term “acoustic-verbal hallucinations” (AVH) because it is broader and more frequently used in the literature to describe the phenomenology of hallucinations in children and adolescents with PTSD. In this population, reported experiences are not limited to imperative or commanding voices. Patients may also describe hearing a voice calling them by their first name, speaking to them in a neutral tone, or voices talking to each other, without giving orders. Using the broader term AVH therefore better reflects the clinical diversity observed in our sample and aligns with current research terminology (e.g., Waters et al., 2012; McCarthy-Jones et al., 2014; Jardri et al., 2014).

- **Waters, F., et al.** (2012). Auditory hallucinations in schizophrenia and nonschizophrenia populations: A review and integrated model of cognitive mechanisms. Schizophrenia Bulletin, 38(4), 683–693.
- **McCarthy-Jones, S., et al.** (2014). Hallucinations in children and adolescents: An updated review and practical recommendations for clinicians. Schizophrenia Bulletin, 40(Suppl_4), S703–S715.
- **Jardri, R., et al.** (2014). The neurodynamic organization of modality-dependent hallucinations. Cerebral Cortex, 23(5), 1108–1117.

3. Baseline differences

*Regarding the statement that “no difference was found in emotional and social cognition at baseline,” does this refer to no between-group differences? Since the mean scale scores were not reported, I wonder whether both groups (being PTSD patients) showed similarly impaired emotional and social cognition at baseline; and perhaps only after six months did group differences emerge due to improvement in some individuals?*

We thank the reviewer for this request for clarification. Yes, at baseline, social cognition scores measured with the NEPSY-II (Theory of Mind and Affect Recognition subtests) and emotional cognition scores measured with the Differential Emotions Scale were equivalent between the “case” and “control” groups. We have clarified this point in the manuscript to avoid any ambiguity with the following sentence:

“At T0, social cognition scores measured with the NEPSY-II (Theory of Mind and Emotion Recognition subtests) and emotional cognition scores measured with the DES-IV did not differ significantly between the ‘case’ and ‘control’ groups.”

Furthermore, as suggested, we have specified in the Results section the between-group comparison at baseline for the variables of interest by adding the following sentence:

“Participants with AVH at T1 had at T0, significantly higher malevolence scores in voices (BAVQ-R) and guilt levels (DES IV) compared with those without AVH at T1 (malevolence: p = 0.04; guilt: p < 0.05). Both malevolence and guilt scores at T0 were also significantly correlated with the presence of AVH at T1 (malevolence: r = 0.36, p = 0.04; guilt: r = 0.35, p < 0.05).”

4. Definition of peer bullying

*Given that peer bullying constituted a relatively high proportion of trauma types in the sample, I would appreciate clarification on how "peer bullying" was defined in this study.*

We thank the reviewer for this request for clarification. We have modified the corresponding sentence in the manuscript to indicate that the types of trauma were categorized based on the participant’s self-report during the clinical interview. The revised sentence now reads:

“Fig 2 presents the types of trauma experienced by the participants, based on their self-report during the clinical interview (Fig 2).”

In our study, “peer bullying” refers to any situation in which the participant reported feeling threatened or intimidated by others (children, adolescents, or adults) in their school or social environment. This definition is consistent with the commonly accepted description of bullying as intentional, repeated aggressive behavior occurring in a context of power imbalance (Olweus, 1993).

5. Typographical error in Figure 3

*In Figure 3, there appears to be a typographical error at T0: the label “control group” should be “AVH–.”*

We thank the reviewer for noticing this. The label has been corrected accordingly.

## **Reviewer #2**

1. Study objectives wording

*The current wording “The primary objective of our study” and “The secondary objectives of our study” may imply that the secondary objectives are unrelated. However, the listed secondary objectives appear to elaborate on and further specify the primary objective. Therefore, I suggest revising “The secondary objectives of our study are” to “Specifically, we aim to” or “To this end, we further aim to”, which would better reflect their relationship.*

We thank the reviewer for this helpful suggestion regarding the formulation of our study objectives. We have revised the paragraph to ensure that the secondary objectives are clearly presented as an elaboration of the primary objective. The paragraph in the manuscript now reads as follows:

“The primary objective of our study is to compare emotional and social cognition markers in a population of children and adolescents with PTSD, between those presenting with AVH and those without AVH. Specifically, we aim to:

1. Compare emotional and social cognition markers in a population of children and adolescents with PTSD, between those presenting with persistent auditory AVH at 6-month follow-up and those without AVH at 6 months;
2. Assess the correlations between emotional and social cognition markers and the presence of AVH at baseline and their persistence at 6-month follow-up;
3. Examine the associations between emotional and social cognition markers and the clinical evolution of PTSD between baseline and 6-month follow-up;
4. Investigate the associations between emotional and social cognition markers and the emergence of a psychotic disorder at 6-month follow-up.”
2. Trauma exposure comparison

*Although all participants met the DSM-5 criteria for PTSD, was there a comparison between the AVH and non-AVH groups in terms of the nature or severity of the traumatic events they experienced? Were the stressors similar across groups, or were there meaningful differences? Additionally, did the two groups differ in the time interval between the occurrence of the stressful event and their inclusion in the study? The current mental state of children and adolescents may also be influenced by how much time has passed since the traumatic event.*

We thank the reviewer for raising this important question. Indeed, in our study we did not compare the AVH+ and AVH– groups in terms of the objective nature or severity of the traumatic events they experienced. Our inclusion criterion was a DSM-5 PTSD diagnosis, emphasizing the participant’s subjective experience of trauma rather than an external judgment of one event’s severity over another. This approach aligns with contemporary conceptualizations that highlight the primacy of the individual’s appraisal and experienced impact of trauma as key determinants of PTSD development, irrespective of the objective severity of the event. For instance, recent research underscores that “by focusing on traumatic experiences rather than just traumatic events, it is possible to consider a wider range of situations that may trigger post-traumatic symptoms” (Rossi and al., 2024). Additionally, we did not collect data on the time elapsed between the traumatic event and study inclusion, as many participants reported complex or repeated trauma exposures over time, with decompensation sometimes following a later event rather than the initial one. We now acknowledge this as a limitation in the Discussion.

We have added the following paragraph to the Discussion section of the manuscript:

« This study did not include a comparison between the AVH+ and AVH– groups regarding the objective nature or severity of the traumatic events, as our inclusion criterion was based on meeting DSM-5 diagnostic criteria for PTSD, which emphasizes the subjective experience of trauma (107). In addition, no data were collected on the exact time elapsed between the traumatic event and study inclusion, particularly as many participants reported complex or repeated trauma exposures over time, and PTSD decompensation could occur following a later event rather than the initial one. These factors should be considered as limitations of the present study”.

3. Diagnostic validity and assessor qualifications

*Diagnostic accuracy is critical. Who administered the K-SADS-PL and the MINI-Kid (Mini International Neuropsychiatric Interview for Children and Adolescents)? Did these assessors receive formal training or hold any relevant professional licenses or certifications?*

We thank the reviewer for this question. All clinical interviews and psychometric assessments, including the K-SADS-PL and the MINI-Kid, were conducted by the same evaluator, a child and adolescent psychiatrist and PhD student. This ensured both clinical expertise in administering these instruments and consistency across all evaluations in the study. We have clarified this point in the Methods section, which now states:

“All clinical interviews and psychometric assessments, including the K-SADS-PL, MINI-Kid, WISC-IV, NEPSY-II, BAVQ-R, and DES-IV, were administered by the same evaluator, a child and adolescent psychiatrist and PhD student, ensuring both clinical expertise and consistency across all assessments.”

4. WISC-IV administration

*Who administered the WISC-IV? Did the examiners receive standardized training or hold professional certification to ensure the reliability and validity of the assessment?*

We thank the reviewer for this question. In our study, an abbreviated version of the WISC-IV was administered, consisting of four subtests (one per index): Similarities for the Verbal Comprehension Index, Picture Concepts for the Perceptual Reasoning Index, Digit Span for the Working Memory Index, and Symbol Search for the Processing Speed Index. The purpose of this abbreviated assessment was not to provide a precise full-scale IQ score but rather to screen for intellectual disability (Full Scale IQ < 70) as a non-inclusion criterion. This short-form WISC-IV has been validated for screening purposes, showing good sensitivity and specificity in detecting intellectual disability (e.g., [reference to add]). IQ scores were not included among the variables compared or correlated in the study objectives. All WISC-IV assessments were conducted by the same evaluator, a child and adolescent psychiatrist and PhD student, who had been specifically trained in test administration by the service’s neuropsychologists.

We have clarified this point in the Methods section, which now states:

"French version of the abbreviated form of the WISC-IV (Similarities, Picture Concepts, Digit Span, Symbol Search) (56) was administered to screen for intellectual disability (Full Scale IQ < 70) as a non-inclusion criterion; this short form has demonstrated good sensitivity and specificity for screening purposes. All assessments were conducted by the same evaluator trained in test administration by the service’s neuropsychologists."

5. Psychometric properties

*It would strengthen the study to include more detailed information on the reliability and validity of each test used, particularly those assessing emotional and social cognition.*

We thank the reviewer for this valuable comment. In response, we have added details on the psychometric properties of the instruments used to assess emotional and social cognition. Specifically, the NEPSY-II Theory of Mind and Affect Recognition subtests have demonstrated acceptable convergent and discriminant validity, with moderate reliability for Theory of Mind and lower reliability for Affect Recognition (Korkman et al., 2007; Schworer et al., 2021). The Differential Emotions Scale (DES-IV) has shown adequate construct validity and acceptable internal consistency for assessing discrete emotional experiences in children and adolescents (Izard, 1991; Izard et al., 1993). Finally, the Beliefs About Voices Questionnaire–Revised (BAVQ-R) has demonstrated good internal consistency (mean Cronbach’s α ≈ 0.86 across subscales) and satisfactory psychometric properties in both its original version (Chadwick et al., 2000) and the French validation study (Zanello & Badan Bâ, 2016).

In the revised manuscript, we have therefore included the following modifications in the Methods section:

For the NEPSY-II: “The NEPSY-II Theory of Mind and Affect Recognition subtests have shown acceptable validity and reliability in pediatric populations (71,72) ».

For the Differential Emotions Scale (DES-IV): “The DES-IV has demonstrated adequate construct validity and acceptable internal consistency for assessing discrete emotions in children and adolescents (63,64) ».

For the BAVQ-R: “The BAVQ-R has shown good internal consistency and satisfactory psychometric properties in both its original (66) and French validated version (68) ».

6. Literature support for null findings

*The statement “This study did not find significant results for markers of social cognition, contrary to data reported in the scientific literature” implies a contradiction with previous findings. Could the authors cite specific studies or provide references to support this comparison?*

We thank the reviewer for this helpful comment. We agree that the original wording could suggest a contradiction with previous findings without sufficient clarification. To address this, we have revised the paragraph to provide a more balanced presentation of the literature, citing specific studies that have reported impairments in social cognition as well as those that did not retain social cognition as a consistent vulnerability marker.

The paragraph in the Discussion section has therefore been modified as follows:

“In our study, no significant differences were observed for social cognition markers. Previous studies, however, have reported impairments in social cognition among youth at risk of psychosis and in PTSD populations, suggesting a potential mediating role of social cognition between trauma exposure and subsequent psychopathology, even before the onset of the first psychotic symptoms (46,47,108,109). At the same time, other studies have not retained social cognition as a consistent vulnerability marker for psychotic symptomatology (110), supporting instead the hypothesis of neurodevelopmental differences in social cognition between individuals progressing to schizophrenia spectrum disorders and those experiencing only isolated psychotic symptoms (111,112)”.

7. IQ differences between groups

*The study reports no significant differences in emotional and social cognition between AVH and non-AVH groups at T0. Given that IQ was measured, were there any differences in IQ between these two groups that might account for the findings?*

We thank the reviewer for this important comment. As detailed in our previous response, an abbreviated version of the WISC-IV was administered in this study, including four subtests (Similarities, Picture Concepts, Digit Span, Symbol Search). The Full Scale IQ derived from this abbreviated form was used exclusively as a screening tool to exclude participants with intellectual disability (FSIQ < 70) as a non-inclusion criterion. Consequently, IQ scores were not included as variables to be compared or correlated with emotional or social cognition markers. Moreover, given the limitations in reliability of abbreviated IQ estimates, such scores would not provide a sufficiently valid basis for group comparisons.

To clarify this point, we have added the following sentence to the *Methods* section of the manuscript, in addition to the modifications already made:

“The IQ estimate obtained from this abbreviated form was used exclusively for screening and was not included in group comparisons or correlations.”

8. Non-PTSD comparison group

*The study hypothesizes that AVH emerging after PTSD may be due to impairments in emotional and social cognition, which affect individuals’ interpretations of internal neural signals. However, to support this hypothesis, it may be necessary to include a matched comparison group without PTSD. If both the AVH and non-AVH groups show lower emotional and social cognition scores than the control group—despite all three groups having similar IQs—it would provide a stronger foundation for attributing AVH to post-traumatic deficits in emotional and social cognition. If such a comparison is not currently available, it is recommended to address this as a limitation of the study.*

We thank the reviewer for this insightful comment. We fully agree that including a matched comparison group without PTSD would provide additional support to the hypothesis. In fact, a pilot study using the same methodology and assessments (Dumas et al., *L’Encéphale*, 2022) compared children and adolescents with and without AVH, but without PTSD. In this pilot work, no significant differences in social and emotional cognition markers were observed at baseline, but significant between-group differences emerged at 6 months, which is consistent with the findings of the present study.

In our current sample, we also observed that participants who did not meet DSM-5 PTSD criteria at baseline but presented with persistent AVH at 6 months did meet PTSD diagnostic criteria at follow-up. This supports the idea of a dynamic interplay between trauma exposure, PTSD, and the persistence of AVH over time, whereby persistent AVH may be associated with the later clinical emergence or consolidation of PTSD symptomatology, rather than reflecting exposure to an additional traumatic event.

We have therefore emphasized in the manuscript that the main message of this study is to propose a psychopathological framework. To clarify this point, we have added the following sentence to the Discussion:

“These findings may suggest a psychopathological framework: in children and adolescents, the presence of AVH could warrant systematic exploration of trauma history, as traumatic experiences may contribute to the content of AVH. Conversely, when PTSD is diagnosed, close monitoring may help identify the possible emergence of perceptual disturbances such as hallucinations, or even progression toward a psychotic disorder. This proposed framework is in line with literature suggesting that psychiatric symptoms often evolve dynamically over time (102,103), gradually leading to comorbid conditions (22,104) and supporting a network-based understanding of psychopathology (105,106).”
